# FOXO3-dependent apoptosis limits alcohol-induced liver inflammation by promoting infiltrating macrophage differentiation

**DOI:** 10.1038/s41420-017-0020-7

**Published:** 2018-02-13

**Authors:** Zhuan Li, Jie Zhao, Shujun Zhang, Steven A. Weinman

**Affiliations:** 1 Department of Internal Medicine, University of Kansas Medical Center, Kansas City, KS USA; 2 Chongqing Key Laboratory of Infectious Diseases and Parasitic Diseases, Department of Infectious Diseases, First Affiliated Hospital of Chongqing Medical University, Chongqing, China; 3 Liver Center, University of Kansas Medical Center, Kansas City, KS USA

## Abstract

Alcohol consumption is generally well tolerated by the liver but in some individuals it results in persistent inflammation and liver disease. The mechanisms that regulate alcohol-induced liver inflammation are poorly understood. The transcription factor FOXO3 has previously been shown to be involved in suppressing alcohol-induced liver injury. In this study we demonstrate that in response to alcohol, approximately 10% of mouse hepatic macrophages undergo FOXO3-dependent apoptosis. By 3 days of alcohol exposure total hepatic macrophage numbers declined by 30% but these were restored to normal after 10 days of continued exposure. Whole body or myeloid specific *Foxo3*^-/-^ mice failed to show this apoptotic response. After 10 days of alcohol exposure, *Foxo3*^−/−^ mice had an increased basal inflammatory phenotype and an increase in the proportion of pro-inflammatory CD11b^+^, Ly6C^+^ infiltrating macrophages (IMs) infiltrating. This led to marked sensitivity to LPS with a 5-fold ALT elevation and liver injury after LPS challenge in *Foxo3*^−/−^ but not WT mice. Restoring the early macrophage apoptosis burst with a pulse of intravenous GdCl_3_ at day 2 had no effect on the day 10 phenotype of WT mice but it corrected the hyper-inflammatory phenotype in *Foxo3*^*−/−*^ mice. In conclusion, FOXO3-dependent hepatic macrophage apoptosis in response to ethanol serves to promote differentiation of infiltrating macrophages thus limiting the magnitude of the inflammatory response to ethanol.

## Introduction

Alcoholic liver disease (ALD) is a leading cause of liver-related morbidity and mortality^1,2^. The pathogenesis of ALD is complex but it involves an ethanol-induced release of bacterial products from the intestine and a subsequent intrahepatic inflammatory cascade that interacts with a liver already altered by other effects of ethanol exposure^3^. This can progress to produce cirrhosis and/or liver failure^4^. ALD occurs only in a minority of heavy drinkers^5^ with the severe inflammatory manifestation of ALD, acute alcoholic hepatitis (AH), occurring even less frequently^6^. These observations suggest that the normal liver possesses protective mechanisms that minimize the inflammatory response and generally allow it to tolerate ethanol with minimal consequences. ALD and AH appear to occur upon failure of these protective mechanisms.

Hepatic macrophages are critical to the initiation and maintenance of the inflammatory state induced by alcohol^7^. They are a heterogeneous population consisting of Kupffer cells, yolk sac derived resident macrophages that have a largely anti-inflammatory and tissue surveillance phenotype, and infiltrating monocyte-derived macrophages (IMs) that carry out diverse pro- and anti-inflammatory functions^8^. Several investigators have previously observed the transient loss of hepatic resident macrophages in mice shortly after exposure of mice to ethanol^9–11^. During continued ethanol consumption, total hepatic macrophage numbers recover but there is a change of hepatic macrophage populations consisting of a disappearance of Kupffer cells, arrival of pro-inflammatory IMs and subsequent differentiation of these IMs to a more anti-inflammatory tissue repair phenotype^12^. The mechanisms underlying this anti-inflammatory shift of the macrophage population remain elusive but multiple studies have shown that it can be triggered by the presence of apoptotic bodies^8, 13–15^.

We have previously demonstrated that the transcription factor FOXO3 protects the liver from ethanol-induced inflammation in mice^16^. We further demonstrated that either ethanol or LPS causes phosphorylation of FOXO3 at S-574 which induces apoptosis in monocytes and macrophages^17^. In the current study we sought to determine whether FOXO3 orchestrates a macrophage apoptosis response to ethanol in vivo, and whether this apoptosis plays a role in modulating the hepatic inflammatory phenotype by inducing macrophage phenotype changes. The results show that ethanol feeding of mice results in FOXO3-dependent hepatic macrophage apoptosis. In the absence of FOXO3, this apoptosis process fails to occur and the liver subsequently adopts a more pro-inflammatory macrophage phenotype. Restoring a transient pulse of macrophage apoptosis the day after ethanol exposure significantly attenuated liver inflammation 10 days later in the ethanol-fed *Foxo3*^*−/−*^ mice making them similar to WT mice. These results suggest that FOXO3 serves to limit ethanol-induced inflammation by inducing hepatic macrophage apoptosis with the apoptotic macrophages acting as a signal that subsequently promotes anti-inflammatory differentiation of intrahepatic macrophages.

## Results

### Acute ethanol gavage induces FOXO3-dependent Kupffer cell apoptosis

**Fig. 1:**
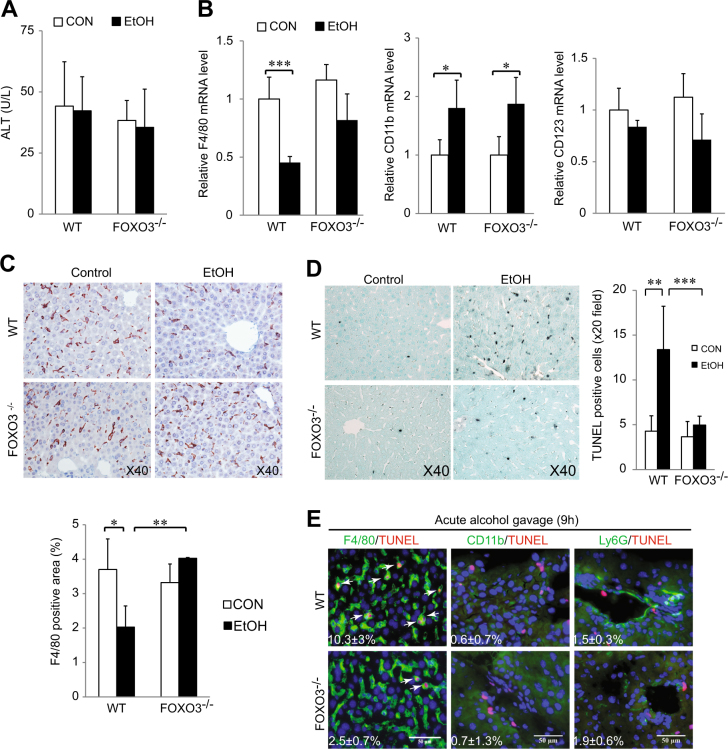
Acute ethanol gavage causes FOXO3-dependent Kupffer cell apoptosis. **a** Serum ALT levels from wild-type (WT) and *Foxo3*^*−/−*^ mice were gavaged with ethanol (5 g/kg). *n* = 4–10. **b** Real time RT-PCR analyses of Kupffer marker F4/80, the infiltrating macrophage marker CD11b, the dendritic cell marker CD123, and the cytokines (TNF-α, IL-6, MCP-1, and IL-10). *n* = 3–5. **c** Representative F4/80 stained liver sections from indicated group. Quantification of F4/80 is shown as a percentage of total area. *n* = 3–5. **d** Representative TUNEL assay in liver sections. **e** Co-staining of TUNEL and myeloid cell markers in liver sections from mice that received ethanol gavage. Arrows indicate double positive cells. Numbers indicate the percent of cells positive for the given marker (F4/80, CD11b or Ly6G) that were also TUNEL positive. *n* = 3. All data were statistically analyzed with two-tailed Student’s *t* test, and presented as mean ± SEM. *P < 0.05, **P < 0.01, ***P < 0.001

Ethanol administration to mice has been reported to induce hepatic macrophage apoptosis^9^ and transiently decrease macrophage number^10, 11^ and we have previously demonstrated that LPS induces FOXO3-dependent cell death in the THP-1 monocyte cell line^17^. We thus tested whether ethanol induced liver macrophage apoptosis was FOXO3-dependent. WT or *Foxo3*^*−/−*^ mice were gavaged with ethanol (5 g/kg) and sacrificed 9 h later. This acute gavage protocol did not change serum alanine aminotransferase (ALT) activity (Fig. 1a) but, similar to previous reports^11^, it reduced the mRNA level of the hepatic macrophage maker F4/80 in WT mice. This effect was absent in *Foxo3*^*−/−*^ mice (Fig. 1b). Immunohistochemical (IHC) staining confirmed that acute ethanol gavage significantly reduced F4/80 positive cells in WT mice (*p* < 0.05) but not *Foxo3*^*−/−*^ mice (Fig. 1c). Ethanol gavage elevated the infiltrating macrophage marker CD11b^18^ but had no effect on a dendritic cell marker CD123^19^ in both WT and *Foxo3*^*−/−*^ mice (Fig. 1b). There was a marked increase of TUNEL positive cells in WT (*p* < 0.01) but not *Foxo3*^*−/−*^mice after acute gavage (Fig. 1d). Co-staining with myeloid cell markers indicated that TUNEL^+^ cells were predominantly F4/80^+^ and there was no colocalization with either brightly staining CD11b+cells (infiltrating macrophages) or Ly6G+cells (neutrophils). There were few detectable CD11b^+^ cells in the liver by 9 h, suggesting that Kupffer cells were the main population responsible for the macrophage decrease after acute gavage. Approximately 10% of the F4/80^+^ cells underwent apoptosis in WT mice (Fig. 1e).

### Myeloid cell FOXO3 is critical for ethanol induced macrophage apoptosis

**Fig. 2:**
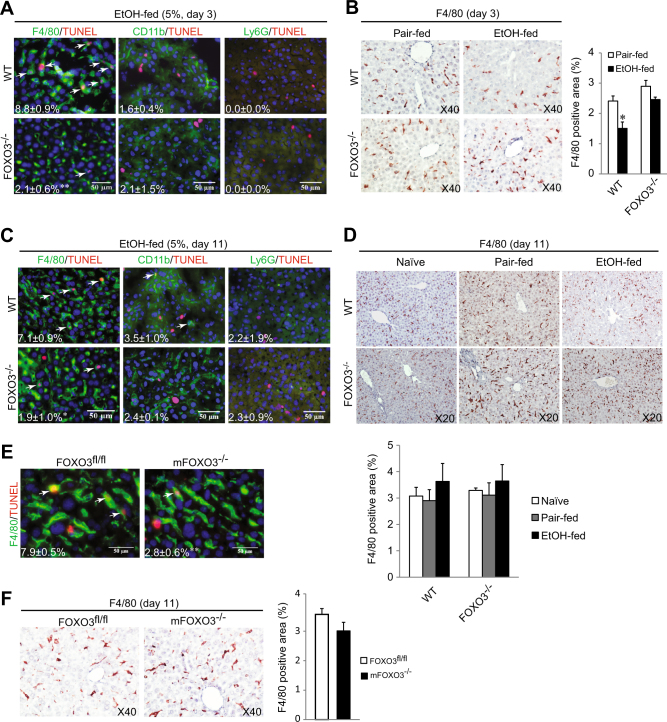
Myeloid FOXO3 is response for alcohol-induced macrophage apoptosis. **a**–**d** WT and *Foxo3*^*−/−*^ mice were fed either control (Pair-fed) or alcohol (EtOH-fed) diet. a Co-staining of TUNEL and myeloid cell markers after 2 days of ethanol. Arrow indicates double positive cells. Numbers indicate percent TUNEL positivity in each cell type. *n* = 3. b IHC staining for F4/80 in liver sections after 2 days of ethanol. Quantification for F4/80 is shown as percentage of total section area. *n* = 3–5. c TUNEL myeloid cell marker co-staining 10 days of ethanol. Arrows indicate double positive cells. Numbers indicate percent TUNEL positivity in each cell type. *n* = 3–4. d IHC staining for F4/80 in liver sections after 10 days of ethanol. Quantification for F4/80 is shown as percentage of total section area. *n* = 3–5. **e–f** LysM cre-Foxo3fl/fl (LysM Foxo3) and matched littermate control mice (Foxo3fl/fl) were pair-fed or fed with ethanol diet for 10 days. e Co-staining of TUNEL and F4/80 in liver sections. Arrows indicate double positive cells. Numbers indicate percent of F4/80 positive cells that were TUNEL positive. *n* = 3. f IHC staining for F4/80 in liver section from mice were fed with ethanol for 10 days. Quantification for F4/80 is showing as percentage of total section area. *n* = 3. All data were statistically analyzed with two-tailed Student’s *t* test, and presented as mean ± SEM. *P < 0.05

We next determined whether voluntary ethanol feeding with a Lieber-DeCarli diet also induces macrophage apoptosis. In WT mice we observed approximately 9 and 7% of F4/80^+^ macrophages undergoing apoptosis after 3 and 11 days of ethanol, respectively (Fig. 2a, c). At 3 days there was an approximately 30% decrease of total F4/80^+^ cells in the liver (Fig. 2b). In *Foxo3*^*−/−*^ mice, in contrast, there was significantly less macrophage apoptosis at both time points (Fig. 2a, b, c). By day 11, the total number of F4/80 positive cells in the liver had returned to baseline in both genotypes (Fig. 2d).

In order to examine whether myeloid FOXO3 itself is critical for ethanol induced macrophage apoptosis, we fed alcohol to m*Foxo3*^*−/−*^ mice and matched littermate controls (*Foxo3*^*fl/fl*^) with the same diet for 10 days. As seen previously, approximately 8% of macrophages in WT mice were TUNEL positive, but similar to the situation in whole body *Foxo3*^*−/−*^ mice, this was reduced to 2.8% in the m*Foxo3*^*−/−*^ mice (Fig. 2e, *P* < 0.01). Macrophage apoptosis did not influence the total number of F4/80 positive cells 10 days after ethanol (Fig. 2f). This data suggests that myeloid FOXO3 is essential for ethanol induced macrophage apoptosis yet in spite of this early apoptosis phase, the total liver macrophage number is able to recover by day 11 of alcohol exposure.

### Loss of FOXO3 exacerbates the pro-inflammatory macrophage phenotype in response to ethanol

**Fig. 3:**
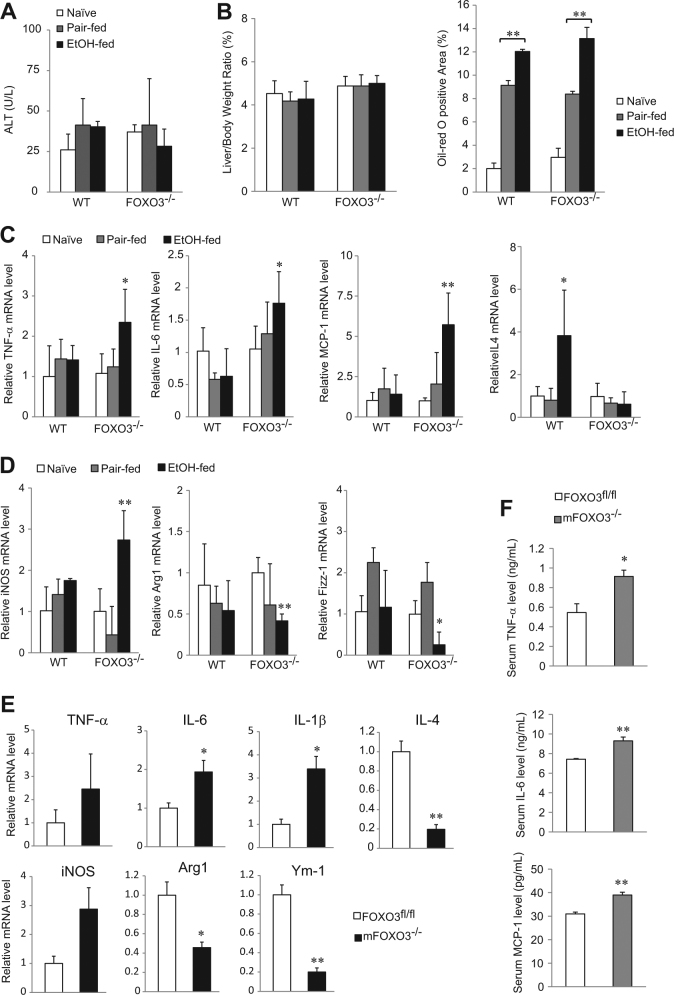
Characteristics of liver phenotype in WT and *Foxo3*^*−/−*^ after 10 days of ethanol feeding. WT and *Foxo3*^*−/−*^ mice were either chow-fed (Naïve), pair-fed or ethanol-fed with the Lieber-DeCarli diet for 10 days and liver injury was evaluated by serum ALT level **a**, liver body ratio and fat accumulation **b** Hepatic mRNA levels of TNF-α, IL-6, MCP-1, IL-4 **c** and the classical macrophage marker iNOS and alternative macrophage markers Arg1 and Fizz-1 **d** were measured by real time RT-PCR. *n* = 3–4. **e**–**f** LysM *Foxo3* and control mice were pair-fed or fed with ethanol diet for 10 days, hepatic mRNA levels of TNF-α, IL-6, IL-1β, IL-4, iNOS and alternative macrophage markers Arg1 and Ym-1 were measured by real time RT-PCR **e** Serum level of TNF-α, IL-6, and MCP-1 were analyzed by ELISA **f** *n* = 3. All data were statistically analyzed with two-tailed Student’s *t* test, and presented as mean ± SEM. *P < 0.05, **P < 0.01

To address the consequences of macrophage apoptosis, we fed mice with an ethanol containing Lieber-DeCarli diet for 10 days and examined the liver phenotype. Not surprisingly, this relatively brief exposure to ethanol did not produce liver injury in either genotype (Fig. 3a) and ethanol equally induced lipid accumulation (Fig. 3b). However, *Foxo3*^*−/−*^ mice displayed a more pro-inflammatory liver phenotype as evidenced by greater mRNA expression of cytokines associated with pro-inflammatory macrophages such as TNF-α, IL-6, and MCP-1, and decreased expression of the anti-inflammatory Th2 cytokine IL-4^20^ (Fig. 3c). 10 days after alcohol exposure, *Foxo3*^*−/−*^ mice had increased liver mRNA expression of the classical macrophage marker iNOS^21^ and decreased alternative macrophage markers arginase 1 (Arg1) and resistin-like molecule alpha 1 (Retnla/Fizz-1)^21^ (Fig. 3d). Similar to whole body *Foxo3*^*−/−*^ mice, m*Foxo3*^*−/−*^ mice also displayed higher mRNA expression of IL-6, IL-1β and iNOS (Fig. 3e) and less expression of IL-4, Arg1 and another alternative macrophage marker, Ym-1^21^. Serum levels of TNF-α, IL-6, and MCP-1 were significantly elevated in m*Foxo3*^*−/−*^ mice (Fig. 3f). Taken together, these data demonstrate increased pro-inflammatory phenotype in the ethanol-fed *Foxo3*^−/−^ and m*Foxo3*^*−/−*^ mice.

### The increased inflammatory phenotype in *Foxo3*^*−/−*^ mice sensitizes the liver to LPS-induced injury

**Fig. 4:**
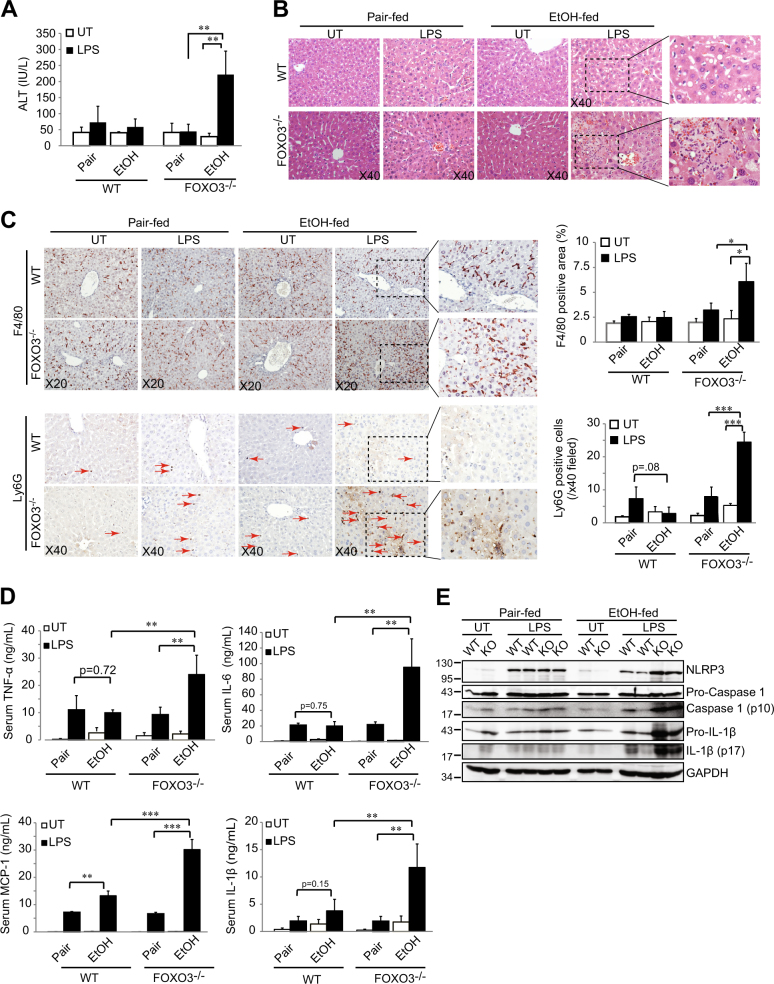
Chronic ethanol feeding sensitizes *Foxo3*^*−/−*^ mice to inflammation and liver injury from LPS. Mice were pair-fed or fed with ethanol for 10 days and thereafter either left untreated (UT) or treated with LPS (10 µg). Mice were sacrificed 6 h after treatment and liver injury was evaluated by serum ALT **a**, and H&E staining **b**. **c** IHC staining for F4/80 and Ly6G in liver sections. Ly6G positive cells are indicated by arrows. Quantification for F4/80 is shown as percentage of total area and Ly6G is shown as total number of Ly6G^+^ cells per field. **d** Serum levels of TNF-α, IL-6, IL-1β, and MCP-1 were evaluated by ELISA. *n* = 4–5. **e** Immunoblots of total hepatic protein from WT or *Foxo3*^*−/−*^ (KO) mice that had been pair-fed or fed with ethanol for 10 days and then either left untreated (UT) or treated with LPS via intraperitoneal injection. All data were statistically analyzed with two-tailed Student’s *t* test, and presented as mean ± SEM. *P < 0.05, **P < 0.01, ***P < 0.001

We next determined whether the ethanol-induced phenotype changes in *Foxo3*^*−/−*^ mice altered inflammatory responses to LPS, a major pathogenic factor in ALD^3^. Mice were fed ethanol for 10 days and then administered a single intraperitoneal dose of LPS (10 µg, 0.5 mg/kg). In the absence of ethanol, LPS had no effect on serum ALT, inflammation, or macrophage or neutrophil infiltration in WT mice or *Foxo3*^*−/−*^ mice (Figs. 4a, b, c). After 10 days of ethanol, however, LPS caused a more than 5-fold increase in serum ALT in *Foxo3*^*−/−*^ mice (Fig. 4a, *p* < 0.01) but had no effect on WT mice. Livers from ethanol-fed *Foxo3*^*−/−*^ mice treated with LPS had large areas of focal inflammation with ballooned, hyper-eosinophilic hepatocytes, hemorrhage, necrosis and macrophage and neutrophil infiltration (Figs. 4b, c). In pair-fed mice, LPS induced increases in TNF-α, IL-6, IL-1β and MCP-1 and these changes were nearly identical in WT and *Foxo3*^*−/−*^ mice (Fig. 4d). Ethanol feeding by itself increased TNFα and IL-1β similarly to the effect of LPS but ethanol did not increase IL-6 or MCP-1. These baseline effects of ethanol in WT and *Foxo3*^*−/−*^ mice were very similar. However, when ethanol feeding was combined with LPS administration, WT and *Foxo3*^*−/−*^ mice behaved quite differently with 2–5 fold greater serum cytokine elevations in the *Foxo3*^*−/−*^ mice (Fig. 4d). Findings of elevated serum IL-1β suggested inflammasome activation in ethanol/LPS treated *Foxo3*^*−/−*^ mice. In the absence of LPS treatment, NLRP3, cleaved caspase-1 and cleaved IL-1β were similar between WT and *Foxo3*^*−/−*^ mice and were not affected by ethanol feeding. LPS treatment of WT mice increased NLRP3 expression without a major change in cleaved caspase-1 or cleaved IL-1β. In ethanol-fed *Foxo3*^*−/−*^ mice, however, LPS treatment resulted in a greater increase in NLRP3 and significantly increased pro-IL-1β, cleaved-caspase-1 and cleaved IL-1β (Fig. 4e). These data demonstrate enhanced LPS-induced inflammasome activation in ethanol-fed *Foxo3*^*−/−*^ mice

.

### IMs are responsible for pro-inflammatory phenotype in ethanol-fed *Foxo3*^*−/−*^ mice

**Fig. 5:**
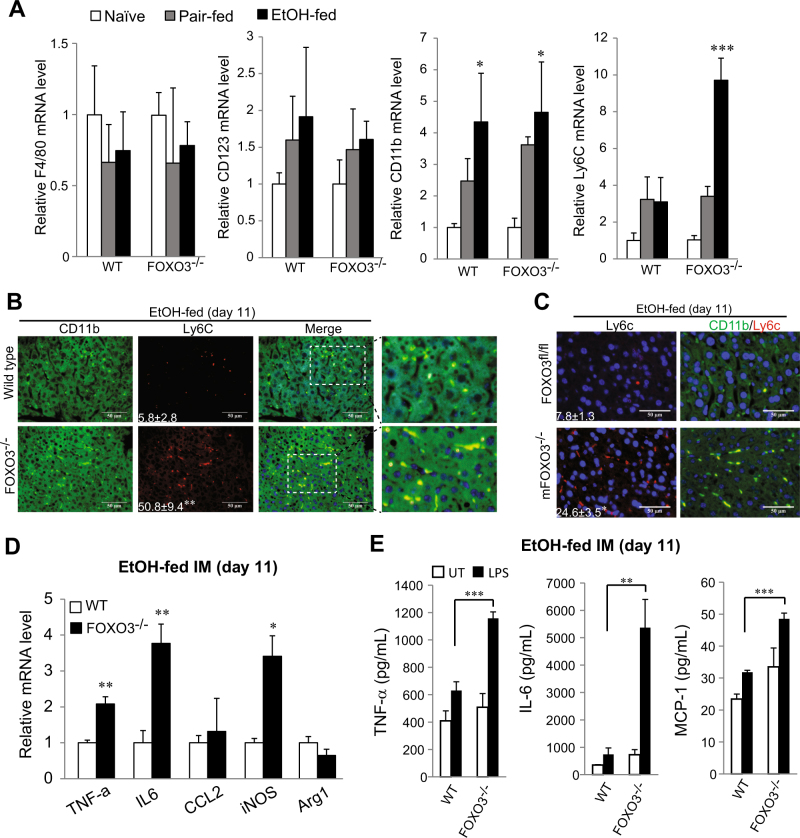
*Foxo3*^*−/−*^ mice displayed higher macrophage-related liver inflammation after 10 days ethanol feeding. **a**–**c** Mice were fed with control or ethanol diet as for Fig. 3. a Hepatic mRNA level of F4/80, CD11b, CD123, and Ly6C was measured by real time RT-PCR. *n* = 3. b Co-staining of Ly6C and CD11b in liver sections. *n* = 3. The right panels are higher magnifications of the merged images. c IHC staining for Ly6C and Co-staining of Ly6C and CD11b in liver sections from LysM *Foxo3* and control mice were pair-fed or fed with ethanol diet for 10 days. *n* = 3. **d**–**e** Mice were fed with control or ethanol for 10 days and CD11b^+^ cells were isolated from the liver, mRNA levels of TNF-α, IL-6, CCl-2, iNOS and Arg1 were measured by real time RT-PCR. *n* = 3. b CD11b^+^ cells as in A were treated with LPS (10 ng/mL) for 6 h. TNF-α, IL-6 and MCP-1 levels were measured by ELISA. *n* = 3. All data were statistically analyzed with two-tailed Student’s *t* test, and presented as mean ± SEM. *P < 0.05, **P < 0.01, ***P < 0.001

We next assessed which cells were responsible for the inflammatory phenotype in *Foxo3*^−/−^ mice. At the end of 11 days of alcohol feeding there were no differences in hepatic F4/80, CD11b, and CD123 mRNA levels between WT and *Foxo3*^*−/−*^ mice (Fig. 5a). However, Ly6C, a marker for pro-inflammatory IMs^22^ was dramatically increased in *Foxo3*^*−/−*^ mice (Fig. 5a, *p* < 0.001). Immunohistochemical staining showed significantly increased Ly6C^+^ cells in *Foxo3*^*−/−*^ mice (Fig. 5b, *p* < 0.01). Co-staining indicated that the Ly6C^+^ cells were IMs as they primarily co-stained with CD11b (Figs 5c, S1A) but not with the T cell marker CD4 (Supplementary Fig. S1B). Similar to *Foxo3*^*−/−*^ mice, m*Foxo3*^*−/−*^ mice also developed a pro-inflammatory phenotype (Figs. 3e, f) with significantly higher Ly6C^+^ cells in the liver compared with control mice after 10 days of ethanol (Fig. 5c, *P* < 0.05).

We next isolated total CD11b^+^ cells from the liver and measured basal and LPS-induced cytokine production. We found that cells from *Foxo3*^*−/−*^ mice had significantly higher basal mRNA expression of TNF-α, IL-6, and iNOS (Fig. 5d). In response to LPS, cells from *Foxo3*^*−/−*^ mice produced significantly more TNF-α, IL-6, and MCP-1 (Fig. 5e). These observations indicate that after 11 days of alcohol exposure, hepatic macrophages from *Foxo3*^*−/−*^ mice have an enhanced inflammatory phenotype with more Ly6C+cells, greater pro-inflammatory cytokine production and increased response to LPS.

### Apoptosis is critical for promoting anti-inflammatory macrophage differentiation

**Fig. 6:**
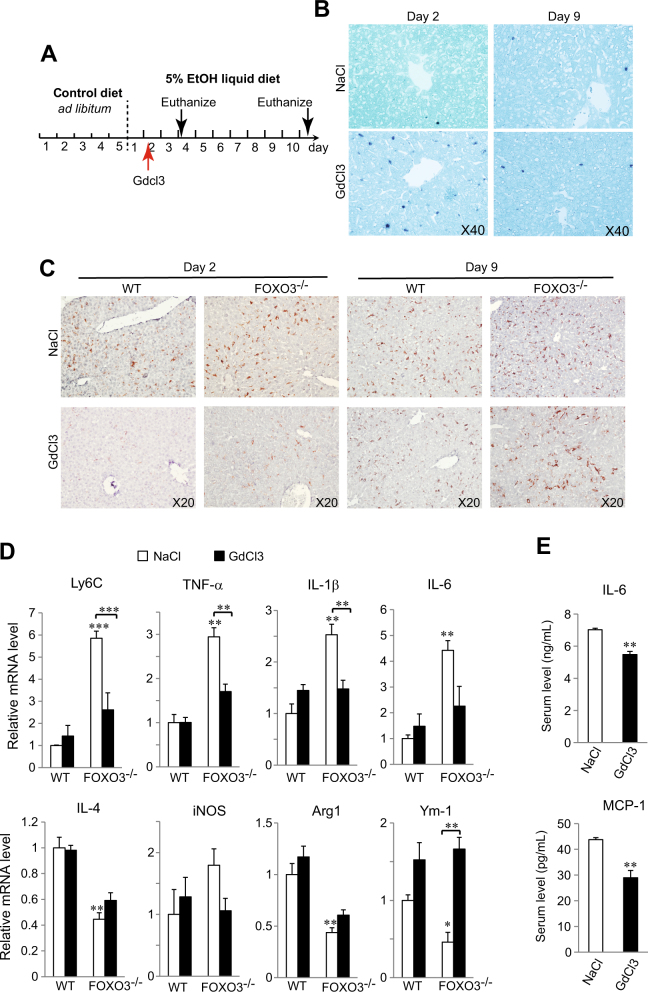
Restoring macrophage apoptosis ameliorates pro-inflammatory phenotype in *Foxo3*^*−/−*^ mice after ethanol feeding. **a** Schematic representation of the time course protocol of the experiment. **b** TUNEL assays from liver sections after injection at indicated times. *n* = 3. **c** IHC staining for F4/80 in liver sections at varies of time as indicated. *n* = 3. **d** Hepatic mRNA levels of Ly6C, TNF-α, IL-1β, IL-6, IL-4, iNOS, Arg1, and Ym-1 at 9 dpi from mice that had received injections of either NaCl or GdCl_3_. *n* = 3. **e** Serum levels of TNF-α, IL-6, and MCP-1 at 9 dpi from *Foxo3*^*−/−*^ mice that received injections of either NaCl or GdCl3. *n* = 3. All data were statistically analyzed with two-tailed Student’s *t* test, and presented as mean ± SEM. *P < 0.05, **P < 0.01

Apoptotic cells are known to interact with phosphatidylserine receptors on macrophages where they synergize with IL4 and other Th2 cytokines to promote differentiation towards an anti-inflammatory tissue repair phenotype^8, 13–15^. We reasoned that the absence of an early apoptosis burst in *Foxo3*^*−/−*^ mice might contribute to the failure of IMs to become more anti-inflammatory over time and thus account for the excess presence of Ly6C^+^ cells. To determine if the loss of macrophage apoptosis explains the persistence of a pro-inflammatory macrophage phenotype we assessed whether a pulse of macrophage apoptosis by a single injection of GdCl_3_^23–25^ could correct the phenotype of *Foxo3*^*−/−*^ mice. We first demonstrated that GdCl_3_ was able to induce macrophage apoptosis independent of the expression of FOXO3 (Supplementary Fig. S2A). Mice were then injected 1 day after ethanol exposure and cell death and inflammatory phenotype was evaluated 2 and 9 days post injection (dpi) (Fig. 6a). By 2 dpi there was a burst of TUNEL positivity of sinusoidal cells and a decrease of liver F4/80 mRNA that was similar in magnitude to that induced by alcohol in WT mice (Supplementary Fig. S2B, compare to Fig. 1b), but by 9 dpi, hepatic macrophage populations had recovered and excess TUNEL-positive sinusoidal cells were no longer detectible (Figs. 6b, c, S2B). In WT mice, GdCl_3_ had no effect on hepatic mRNA expression of cytokines or inflammatory or anti-inflammatory macrophage markers (Fig. 6d). However, in *Foxo3*^*−/−*^ mice, the GdCl_3_ pulse dramatically decreased the inflammatory phenotype at 9 dpi, reducing Ly6C and pro-inflammatory markers, increasing anti-inflammatory markers and essentially correcting the abnormalities seen in the *Foxo3*^*−/−*^ mice (Fig. 6d). We also observed a corresponding decrease in serum cytokine levels (Fig. 6e) suggesting that restoring an early apoptosis burst was able to partially correct the pro-inflammatory phenotype of alcohol-fed *Foxo3*^*−/−*^ mice.

## Discussion

Ethanol consumption has the potential to produce significant liver inflammation and injury through its effects on gut permeability, hepatocyte oxidative stress, and innate immune activation^3^. Nonetheless, most individuals who drink heavily do not develop liver disease, suggesting the existence intrinsic hepato-protection mechanisms. Findings from our lab previously demonstrated that FOXO3 serves as a protective factor for ethanol-induced liver injury^16^ and in response to ethanol, FOXO3 is phosphorylated at S-574 converting it into a factor that causes apoptosis in macrophages^17^. In this study we examined the connection between macrophage apoptosis and the control of liver inflammation. We confirmed that, as observed previously^9^, about 10% of resident hepatic macrophages undergo apoptosis in response to alcohol but total macrophage number is quickly restored. In Foxo3^−/−^ mice, this initial burst of macrophage apoptosis is absent and 10 days later the hepatic macrophage pool has an abnormally pro-inflammatory phenotype. This creates the potential for liver injury in response to an LPS challenge such as might occur as a result of alcohol-induced changes in gut barrier function. Restoring a pulse of macrophage apoptosis, in this case with a single IV injection of GdCl_3_, was able to prevent the development of the pro-inflammatory phenotype in *Foxo3*^*−/−*^ mice.

The process by which the hepatic macrophage pool adapts over the course of alcohol exposure and the importance of this adaptation to controlling liver inflammation is only partially understood. Previous studies have shown that ethanol consumption by mice initially results in the appearance of pro-inflammatory Ly6C^hi^, monocyte-derived macrophages in the liver. Over time these cells differentiate into Ly6C^low^, anti-inflammatory macrophages limiting ethanol-induced inflammation^12^. Cytokines such as IL4 and IL13 as well as apoptotic bodies have long been understood to promote this anti-inflammatory macrophage differentiation but recently it has emerged that the presence of signaling cascades initiated by apoptotic body/receptor interactions are essential in order for IL-4 and IL-13 to promote alternative, anti-inflammatory macrophage differentiation^15^. Macrophage cell death similarly induces anti-inflammatory macrophage differentiation after bacterial infection^8, 26^. Our studies support this concept and demonstrate that apoptosis of resident macrophages supplies a signal that promotes the differentiation of IMs.

Although *Foxo3*^−/−^ mice have an increased inflammatory phenotype after 10-day alcohol exposure, they do not develop spontaneous liver injury. Liver injury occurred only when we challenged these mice with a low, ordinarily nontoxic dose of LPS. Gut-derived LPS plays a critical role in initiating the inflammatory response of the liver to ethanol exposure^27–30^ but is not of itself sufficient to produce liver injury. Injury may only occur when elevated LPS translocation interacts with a state of enhanced hepatic LPS sensitivity.

Our finding that ethanol induces apoptosis of about 10% of intrahepatic macrophages confirms a number of previous findings. Macrophage apoptosis has been observed under many circumstances including during response to bacterial infection^31^, and it can be directly produced by exposure to LPS^32^, particularly under conditions in which NF-κB activation is inhibited^33^. Ethanol-induced intrahepatic macrophage apoptosis has been most definitively shown by Cohen et al.^9^ who did simultaneous TUNEL assays and immuno-histochemical stains which showed that ethanol induces Kupffer cell apoptosis associated with an early spike of pro-inflammatory cytokine expression^10^. Here we identified that FOXO3 is crucial for this ethanol induced macrophage apoptosis. This is consistent with our previous demonstration that ethanol-induced, JNK-dependent phosphorylation of FOXO3 at S-574 triggers monocyte and macrophage apoptosis by induction of pro-apoptotic genes and suppression of Bcl-2^17^.

There have been suggestions that hepatocyte apoptosis occurs in alcoholic hepatitis and that apoptotic bodies derived from hepatocytes may be responsible for macrophage differentiation^12^. It is plausible that FOXO3 might also be responsible for a low level of hepatocyte apoptosis in response to ethanol due to its induction of Bim^34^ and/or PUMA^35^, but in these studies of early liver effects from a relatively low-dose alcohol exposure we did not detect appreciable hepatocyte apoptosis after ethanol and both whole body and myeloid specific *Foxo3*^*−/−*^ mice developed a similar inflammatory phenotype. Nonetheless, we considered the possibility that loss of an apoptotic pathway in *Foxo3*^*−/−*^ hepatocytes might potentiate non-apoptotic hepatocyte death which would contribute liver injury^36^ by converting the more benign apoptosis process into the more pro-inflammatory process of necroptosis. We measured key components of necroptosis, RIPK1, RIPK3 and caspase 8 cleavage in our mice after 3 weeks of ethanol (Supplementary Fig. S3). There were equal increases of RIPK3 expression and caspase 8 activation in both WT and *Foxo3*^*−/−*^ mice (Supplementary Fig. S3A–B) thus suggesting that the phenotype of the *Foxo3*^*−/−*^ mice is not due to increased ethanol-induced hepatocyte necroptosis.

Further evidence supporting a role of FOXO3-dependent myeloid cell apoptosis in adaptation of the liver to alcohol comes from recent studies from our lab showing that alterations of myeloid cell FOXO3 function are present in alcoholic hepatitis patients. Monocytes from patients with alcoholic hepatitis fail to undergo normal FOXO3 phosphorylation and apoptosis in response to LPS^37^. In this case, the defect was attributed to a change in FOXO3 acetylation state. This defect, if present in intrahepatic macrophages as well, could contribute to the unusual sensitivity to ethanol in alcoholic hepatitis patients. Overall, our results are consistent with the following working hypothesis. Under normal conditions, exposure of the liver to ethanol causes JNK-dependent S-574 phosphorylation of FOXO3 and subsequent apoptosis in about 10% of Kupffer cells. These apoptotic bodies interact with pro-inflammatory Ly6C^+^ infiltrating macrophages that enter the liver in response to alcohol and synergize with IL-4 to induce a delayed differentiation of the IMs to a more tissue repair and less inflammatory phenotype. This allows the liver to adapt to alcohol by downregulating the innate immune response.

In conclusion, this study has demonstrated that an early burst of FOXO3-dependent macrophage apoptosis plays a role in the evolution of the inflammatory environment of the liver after ethanol exposure. This effect tends to limit the intrahepatic inflammatory response and may contribute to hepatic ethanol tolerance. In the absence of this FOXO3-dependent mechanism, the liver displays hypersensitivity to LPS-induced injury. Defects in the FOXO3 apoptosis pathway have been observed in circulating monocytes from patients with alcoholic hepatitis^37^ and this supports the importance of this pathway in hepato-protection from ethanol.

## Materials and methods

### Animals and treatments

*Foxo3*^*−/−*^ mice were provided by Dr. Kana Miyamoto (Keio University, Tokyo) and were generated as described^38^. Heterozygotes were bred together, obtaining both knockout and wild-type (WT) littermates. To generate myeloid specific FOXO3 knockout mice (m*Foxo3*^−/−^), Foxo3^fl/fl^ mice with a floxed FOXO3 allele exon 2 (Jackson Labs, Bar Harbor, ME) were backcrossed into the C57BL/6 background for six generations. They were then bred with mice expressing the Cre recombinase under the control of lysozyme 2 gene (Lyz2) promoter/enhancer elements (B6.129P2-Lyz2tm1(cre)Ifo/J, hereafter LysM cre, Jackson Labs). They were used for ethanol feeding experiments at 3 to 6 months of age. All mice were housed in a temperature-controlled, specific pathogen-free environment with 12-h light-dark cycles and fed regular mouse chow and water ad libitum. All animal handling procedures were approved by the Institutional Animal Care and Use Committees at the University of Kansas Medical Center (Kansas City, KS).

We used mice of different genders for specific experiments to be consistent with previous reports^39, 40^. For acute ethanol feeding, female mice were gavaged with ethanol (5 g/kg body weight) in the morning and sacrificed 9 h later^39^. For voluntary ethanol feeding, female and male mice were initially fed the control *Lieber-DeCarli* diet (BioServ, Flemington, NJ) ad libitum for 5 days to acclimatize them to a liquid diet. Then mice were allowed free access to the ethanol *Lieber-DeCarli* diet containing 5% (vol/vol) ethanol for 10 days, and control-fed groups were pair-fed with the isocaloric control diet. For LPS injection, male mice were fed with liquid diet ethanol feeding described above^40^. At the end of study, all mice received a single intraperitoneal injection of 10 μg LPS (*E. coli*, Serotype O55:B5 S-form, Enzo Life Science, Farmingdale, NY) and sacrificed 6 h later. Liver tissue and venous blood were obtained. Serum was stored at −80 °C. Liver samples were fixed in formalin for histological examination or frozen in liquid nitrogen and stored at −80 °C. Total liver lysates were prepared using RIPA buffer [1% NP40, 0.5% sodium deoxycholate, 0.1% sodium dodecyl (lauryl) sulfate].

### Immunohistochemistry

Liver tissue sections (5 μm thick) were prepared from paraffin-embedded samples as previously described^16^. Immunohistochemistry (IHC) was performed by deparaffinization and rehydration, followed by antigen retrieval by heating in a pressure cooker (121 °C) for 5 min. Peroxidase activity was blocked by incubation in 3% hydrogen peroxide for 10 min. Sections were rinsed three times in TBS-T (20 mM Tris, pH 7.6. 150 mM NaCl. 0.1% Tween 20) and incubated in 5% normal goat serum in TBS-T at room temperature for 1 h. After removal of blocking solution, slides were placed into a humidified chamber and incubated with primary antibodies in blocking buffer (3% normal goat serum in PBS) and incubated over night at 4 °C. After washing, slides were covered with SignalStain Boost IHC Detection Reagent (Cell Signaling Technologies, Boston, MA) for 30 min at room temperature. After washing two times with TBS-T, the Substrate-Chromgen Solution (VECTOR NovaRED, Substrate Kit, Vector Laboratories, Burlingame, CA) was applied, slides were incubated 5–10 min and counterstained with Hemtoxylin. For immunofluorescent staining of Ly6C and CD11b, paraffin sections were permeabilized with 0.2% Triton-100 for 20 min and were blocked with protein block (DAKO, Santa Clara, CA) for 15 min followed by overnight incubation with the primary CD11b and Ly6C antibodies (1:200). After washing with PBS (3 times, 5 min each), sections were incubated with Alexa Fluor 488-conjugated goat anti-rabbit IgG or Alexa Fluor 594-conjugated donkey anti-rat IgG (1: 5000; Molecular Probes, Waltham, MA) for 1 h in the dark at room temperature.

### TUNEL staining

Cell death was detected in situ in mouse liver paraffin-embedded sections by enzymatic labeling of DNA strand breaks with a TUNEL assay kit (In Situ Cell Death Detection Kit, Roche, Indianapolis, IN) according to the manufacturer’s instructions, followed by counterstaining with ethyl-[4-[[4-[ethyl-[(3-sulfophenyl) methyl] amino] phenyl]-(4-hydroxy-2-sulfophenyl) methylidene]-1-cyclohexa-2, 5-dienylidene]-[(3-sulfophenyl) methyl] azanium), (Fast Green FCF, Acros Organics, NJ,). Quantification of all TUNEL staining was performed by examining at least five randomly selected fields in each liver section by two investigators who were blinded to sample identity.

For double staining of TUNEL and myeloid cell markers, frozen liver section were mounted on glass slides, fixed with paraformaldehyde and washed in PBS. Sections were then blocked with protein block (DAKO, Santa Clara, CA) for 15 min followed by overnight incubation with the primary F4/80, CD11b or Ly6G antibodies (1:200). After washing with PBS, sections were incubated with Alexa Fluor 594-conjugated goat anti-rabbit IgG or donkey anti-rat IgG (1: 5000; Molecular Probes, Waltham, MA) for 1 h in the dark at room temperature. All sections were additionally stained for apoptotic cells using the in situ Cell Death Detection Kit (Fluorescein, Roche) according to the manufacturer’s instructions. Images were acquired using a Nikon Eclipse Ti microscope (Nikon Americas Inc., Melville, NY).

### Isolation of liver infiltrating macrophages

Infiltrating macrophages were isolated using a multi-step collagenase procedure^41^ and then subjected to centrifugation on a Percoll (Sigma-Aldrich, St. Louis, MO) gradient. In brief, the liver was perfused with calcium-free solution and then digested with a collagenase (Sigma-Aldrich) perfusion. Dispersed cells were released from the isolated liver, and hepatocytes were collected by 50×*g* centrifugation. Non-parenchymal cells were fractionated by Percoll gradient at 1350 g for 10 min and infiltrating macrophages were purified by using CD11b magnetic beads (Miltenyi Biotec, Auburn, CA). For in vitro co-culture with apoptotic macrophages, CD11b^+^ hepatic macrophages were isolated from mice that had been either pair-fed or ethanol-fed for 2 days. Apoptosis was induced by incubating mouse primary peritoneal macrophages^17^ with Etopside (200 µM, Sigma-Aldrich) at 37 °C for 5 h. This preparation yielded > 90% TUNEL-positive apoptotic cells by 5 h and after 24 h there were no detectable mRNA could be isolated from these cells. The hepatic CD11b^+^ cells (5 × 10^5^) were then co-cultured with 5 × 10^5^ apoptotic macrophages for 24 h and mRNA was isolated and used for expression analysis.

### Real-time PCR

RNA was extracted and purified from mouse liver or isolated cells using the RNeasy Mini Kit (Qiagen, Valencia, CA). cDNA was generated with the RNA reverse transcription kit (Applied Biosystems, Warrington, UK). Quantitative RT-PCR was performed in a CFX96 real-time system (Bio-Rad, Hercules, CA) using specific sense and antisense primers in 25 μl reaction volumes containing 12.5 μl SYBR Green PCR master mix (Applied Biosystems), 10.5 μl of 1 μmol/l primer stock and 2 μl cDNA. Primer sequences are presented in Supplementary Table 1.

### Induction of macrophage apoptosis in vivo

Mice received a single intravenous (i.v) injections of GdCl_3_ (25 mg/kg, Sigma-Aldrich) or saline (0.9% NaCl, Baxter Healthcare Co, Deerfield, IL) via tail vein 1 day after ethanol exposure. Macrophage apoptosis was assessed at 2 days post injection.

### Antibodies

Anti-F4/80 (SP115) antibody was purchased from Novus (Littleton, CA). Anti-Ly6G (ab25377), anti-CD11b (ab133357) and anti-Ly6C (ab15627) were purchased from Abcam (Cambridge, MA).

### ALT assay and ELISA measurement

Serum ALTs were measured by commercial kit (TECO Diagnostics, Anaheim, CA). TNF-α, IL-6 levels were measured with ELISA Ready-SET-Go! kits (eBioscience, San Diego, CA) in serum according to the manufacturer’s protocol. IL-1β and MCP-1 were measured with DouSet ELISA kit from R&D Systems (Minneapolis, MN) according to the manufacturer’s protocol.

### Statistical analysis

Data were presented as mean ± standard error of the mean (SEM). Statistical significance between two groups was calculated by 2-tailed unpaired Student’s t-test. Unless otherwise stated, a *P*-value of <0.05 was considered significant.

## Supplementary information

supplementary information
