# Supplementary material for: FOXO3-dependent apoptosis limits alcohol-induced liver inflammation by promoting infiltrating macrophage differentiation
Source: Cell Death Discov. 2018 Feb 13;4:16. doi: 10.1038/s41420-017-0020-7 (PMC5841311; doi:10.1038/s41420-017-0020-7)

***Supplementary* *information***

**FOXO3-dependent apoptosis limits alcohol induced liver inflammation by promoting infiltrating macrophage differentiation**

Zhuan Li^1^, Jie Zhao^1^, Shujun Zhang^1^ and Steven A. Weinman^1,2^

^1^Department of Internal Medicine, and ^2^Liver Center, University of Kansas Medical Center, Kansas City, KS 66160, U.S.A.

***Supplementary Table 1.* Primer sequences used for RT-PCR.**

| F4/80 forward | 5'-CTTTGGCTATGGGCTTCCAGTC-3' |
| --- | --- |
| F4/80 reverse | 5'-GCAAGGAGGACAGAGTTTATCGTG-3' |
| CD11b forward | 5'-AAACCACAGTCCCGCAGAGA-3' |
| CD11b reverse | 5'-CGTGTTCACCAGCTGGCTTA -3' |
| Ly6G forward | 5'-TGCGTTGCTCTGGAGATAGA-3' |
| Ly6G reverse | 5'-CAGAGTAGTGGGGCAGATGG-3' |
| CD123 forward | 5’-CTGGCATCCCACTCTTCAGAT-3’ |
| CD123 reverse | 5’-GGTCCCAGCTCAGTGTGTA-3’ |
| TNFα forward | 5’-AGGCTCTGGAGAACAGCACAT-3’ |
| TNFα reverse | 5’-TGGCTTCTCTTCCTGCACCAAA-3’ |
| IL-6 forward | 5’-TTCCATCCAGTTGCCTTCTT-3’ |
| IL-6 reverse | 5’-CAGAATTGCCATTGCACAAC-3’ |
| MCP1 forward | 5’-GGGCCTGCTGTTCACAGTT-3' |
| MCP1 reverse | 5’-CCAGCCTACTCATTGGGAT-3’ |
| IL-10 forward | 5’-GGTTGCCAAGCCTTATCGGA-3’ |
| IL-10 reverse | 5’-ACCTGCTCCACTGCCTTGCT-3’ |
| CD69 forward | 5'-TGGTCCTCATCACGTCCTTAATAA-3' |
| CD69 reverse | 5'-TCCAACTTCTCGTACAAGCCTG-3' |
| Ly6C forward | 5'-GCAGTGCTACGAGTGCTATGG-3' |
| Ly6C reverse | 5'-ACTGACGGGTCTTTAGTTTCCTT-3' |
| IL33 forward | 5'-AGCTCTCCACCGGGGCTCAC-3' |
| IL33 reverse | 5'-GCCTGCGGTGCTGCTGAACT-3' |
| iNOS forward | 5’-AATCTTGGAGCGAGTTGTGG-3’ |
| iNOS reverse | 5’-CAGGAAGTAGGTGAGGGCTTG-3’ |
| Arg1 forward | 5’-CTCCAAGCCAAAGTCCTTAGAG-3’ |
| Arg1 reverse | 5’-AGGAGCTGTCATTAGGGACATC-3’ |
| Fizz-1 forward | 5'-TCCCAGTGAATACTGAGA-3' |
| Fizz-1 reverse | 5'-CCACTCTGGATCTCCCAAGA-3' |
| Ym-1 forward | 5'-CATGAGCAAGACTTGCGTGAC-3' |
| Ym-1 reverse | 5'-GGTCCAAACTTCCATCCTCCA-3' |
| GAPDH forward | 5'-[CGTCCCGTAGACAAAATGGT](https://www.idtdna.com/orderstatus/SpecSheet.aspx?OrderNum=5423667&MfgID=0&SearchDays=&SearchNum=&SearchPO=&SearchRef=)-3' |
| GAPDH reverse | 5'-[TTGAGGTCAATGAAGGGGTC](https://www.idtdna.com/orderstatus/SpecSheet.aspx?OrderNum=5423667&MfgID=0&SearchDays=&SearchNum=&SearchPO=&SearchRef=)-3' |

**Supplementary Figure 1.** Wild type (WT) and *Foxo3^-/-^* mice were fed with either control Lieber-DeCarli diet (Pair-fed) or Lieber-DeCarli diet containing 5% ethanol (EtOH-fed) for 2 or 10 days. (A) Co-staining of Ly6C (red) with monocyte marker CD11b or (B) Ly6C (red) with T cell marker CD4 (green) in liver sections. Scale bar: 50 µm. n=3 mice per group.


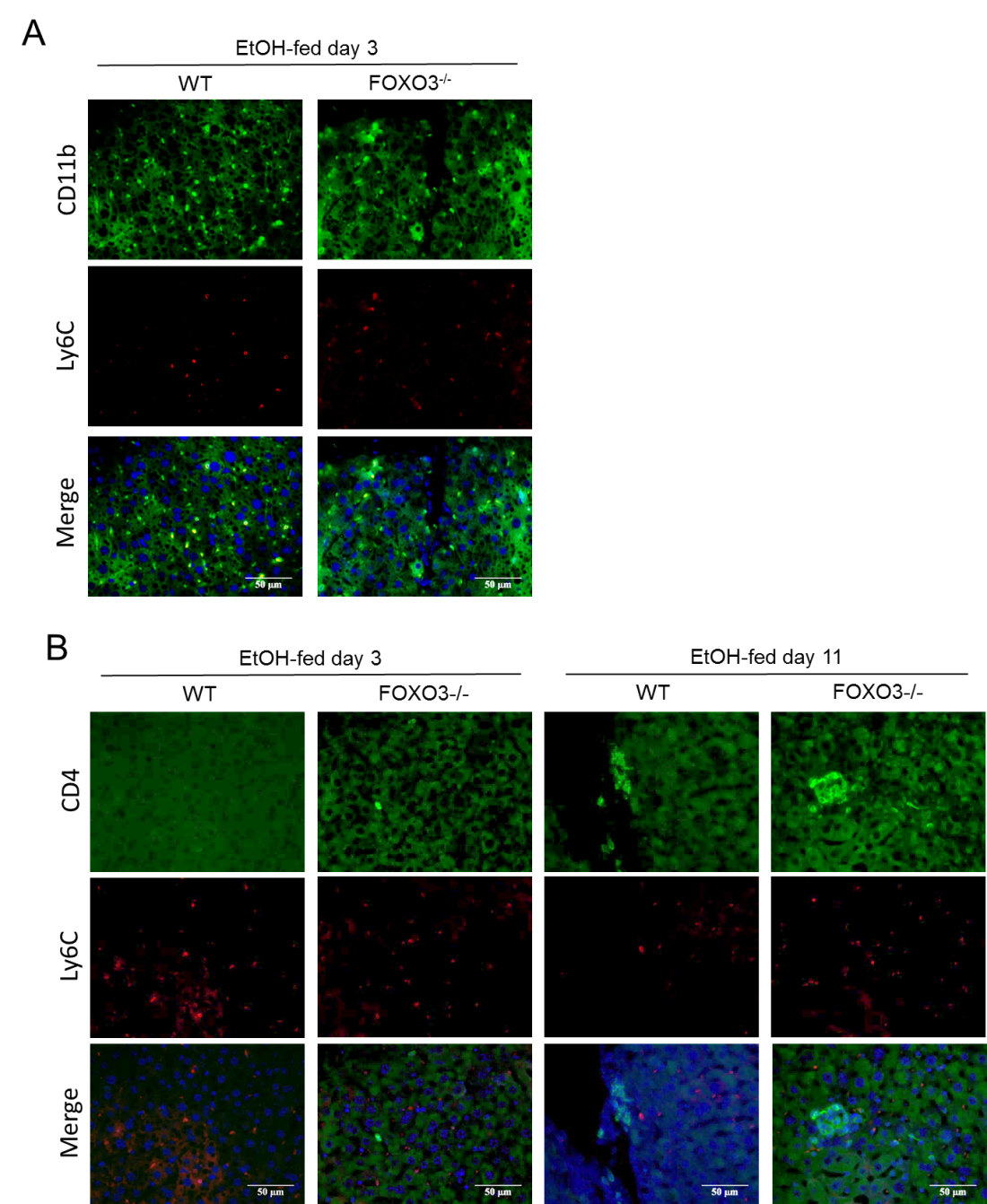


**Supplementary Figure 2.** (A) TUNEL assays with peritoneal macrophages from wild type or FOXO3^-/-^ mice that were treated with saline or GdCl_3_ (270 µM) for 24 hours. Scale bar: 100 µm. n=3 mice per group. Quantification for TUNEL is showing as total TUNEL positive cell number per field. Graph shows mean ± SEM. ***P < 0.001, Student’s t test. (B) Hepatic mRNA levels of F4/80 at either 2 or 9 days post injection from FOXO3^-/-^ mice that had received single injections of either NaCl or GdCl_3_ at day 1 of alcohol feeding. Results were measured by real time RT-PCR normalized to GAPDH. n=3 mice for each group. Graphs show mean ± SEM.


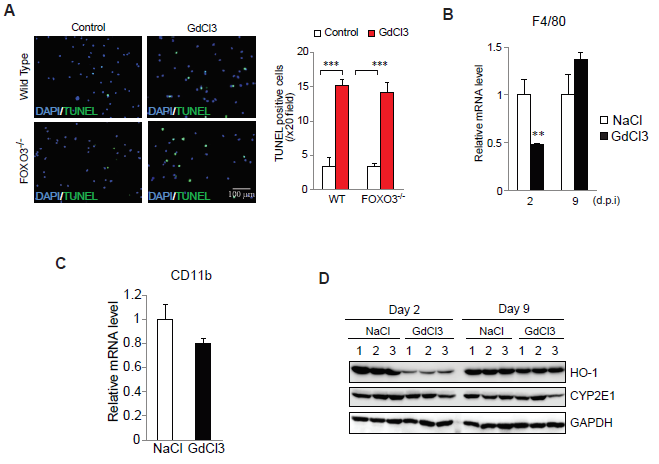


**Supplementary Figure 3.** (A) Representative immunoblots of total hepatic protein from mice either pair-fed or fed with Lieber-DeCarli diet containing ethanol (concentration was increased stepwise to 1.6%, 3.2%, 4.8%, and, finally, 6.4% (v/v)) for 3 weeks. (B) Relative protein levels of RIPK1, RIPK3 and cleaved caspase 8 (C-Caspase 8) from total hepatic protein from mice either Pair-fed or EtOH-fed or 3 weeks. Data shows relative expression assessed by densitometry of immunoblots and normalized to β-Actin. n= 5-15 mice per group. Graph shows mean ± SEM. *P < 0.05, **P < 0.01 , ***P < 0.001, Student’s t test.


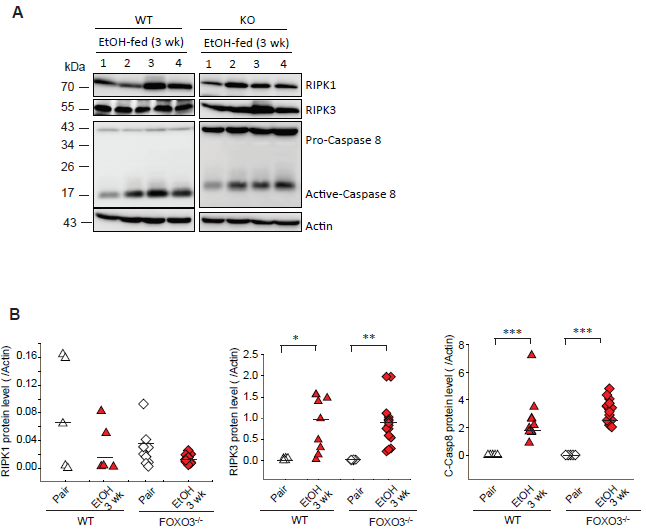

Supplement: Supplementary file 1 — supplementary information [file 41420_2017_20_MOESM1_ESM.docx]
